# Pepsin-pancreatin protein hydrolysates from extruded amaranth inhibit markers of atherosclerosis in LPS-induced THP-1 macrophages-like human cells by reducing expression of proteins in LOX-1 signaling pathway

**DOI:** 10.1186/1477-5956-12-30

**Published:** 2014-05-19

**Authors:** Alvaro Montoya-Rodríguez, Jorge Milán-Carrillo, Vermont P Dia, Cuauhtémoc Reyes-Moreno, Elvira González de Mejía

**Affiliations:** 1 Programa Regional del Noroeste para el Doctorado en Biotecnología, FCQB-UAS, Ciudad Universitaria, AP 1354, Culiacán, Sinaloa CP 80000, México; 2 Food Science and Human Nutrition, University of Illinois at Urbana-Champaign, 228 ERML, MC-051, 1201 West, Gregory Drive, Urbana, IL 61801, USA

**Keywords:** Atherosclerosis, Amaranth, THP-1, Hydrolysates, Macrophages

## Abstract

**Background:**

Atherosclerosis is considered a progressive disease that affects arteries that bring blood to the heart, to the brain and to the lower end. It derives from endothelial dysfunction and inflammation, which play an important role in the thrombotic complications of atherosclerosis. Cardiovascular disease is the leading cause of death around the world and one factor that can contribute to its progression and prevention is diet. Our previous study found that amaranth hydrolysates inhibited LPS-induced inflammation in human and mouse macrophages by preventing activation of NF-κB signaling. Furthermore, extrusion improved the anti-inflammatory effect of amaranth protein hydrolysates in both cell lines, probably attributed to the production of bioactive peptides during processing. Therefore, the objective of this study was to compare the anti-atherosclerotic potential of pepsin-pancreatin hydrolysates from unprocessed and extruded amaranth in THP-1 lipopolysaccharide-induced human macrophages and suggest the mechanism of action.

**Results:**

Unprocessed amaranth hydrolysate (UAH) and extruded amaranth hydrolysate (EAH) showed a significant reduction in the expression of interleukin-4 (IL-4) (69% and 100%, respectively), interleukin-6 (IL-6) (64% and 52%, respectively), interleukin-22 (IL-22) (55% and 70%, respectively). Likewise, UAH and EAH showed a reduction in the expression of monocyte-chemo attractant protein-1 (MCP-1) (35% and 42%, respectively), transferrin receptor-1 (TfR-1) (48% and 61%, respectively), granulocyte-macrophage colony-stimulating factor (GM-CSF) (59% and 63%, respectively), and tumor necrosis factor-α (TNF-α) (60% and 63%, respectively). Also, EAH reduced the expression of lectin-like oxidized low-density lipoprotein receptor-1 (LOX-1) (27%), intracellular adhesion molecule-1 (ICAM-1) (28%) and matrix metalloproteinase-9 (MMP-9) (19%), important molecular markers in the atherosclerosis pathway. EAH, led to a reduction of 58, 52 and 79% for LOX-1, ICAM-1 and MMP-9, respectively, by confocal microscopy.

**Conclusions:**

Extruded amaranth hydrolysate showed potential anti-atherosclerotic effect in LPS-induced THP-1 human macrophage-like cells by reducing the expression of proteins associated with LOX-1 signaling pathway.

## Background

Cardiovascular disease (CVD) is the principal cause of death around the world, including coronary heart disease, rheumatic heart disease, heart failure, hypertension, hyperlipidemia, congenital heart disease, and atherosclerosis [1]. There is a strong relationship between CVD and the diet [2]. The consumption of whole cereals, fruits and vegetables could help to reduce the risk to develop diseases such as obesity, diabetes, hypertension, which are related with the development of CVD, specifically atherosclerosis [3]. Atherosclerosis is considered as a progressive disease derived from endothelial dysfunction and inflammation, with the progressive occlusion of the arteries by a plaque, which consist of cholesterol, aggregating proteins, calcium and other substances [4-7]. Also, atherosclerosis was regarded as a bland lipid storage disease, but nowadays, it is known that it also involves an ongoing inflammatory response [8]. Chronic inflammation is involved in the initiation and progression of atherosclerosis, and plays an important role in thrombotic complications [3,9,10]. A rupture of the plaque results in the total occlusion of the artery, leading to obstruction of blood flow; hence a heart attack occurs [11]. Atherosclerosis mainly affects the arteries that bring blood to the heart (coronaries), to the brain (carotid, cerebral and vertebral) and to the lower end (iliac and femoral) [12]. When an erosion, fissure or rupture of the plaque occurs, it leads to the thrombus formation, causing complications such as cerebrovascular disease, heart disease or peripheral arterial disease [13].

Diet is strongly related with initiation and progression of CVD, since an increment in the calories intake (diet rich in cholesterol and saturated fat) can elevate the serum level of low-density lipoproteins (LDLs) [6,10]. When the LDLs are oxidized in the sub-endothelial environment, by different factors such as free radicals, it is the triggering step to the atherogenic process [10]. Oxidized LDL (ox-LDL) plays an important role in the atherosclerotic process. When ox-LDL binds to its principal receptor, lectin-like oxidized low-density lipoprotein receptor 1 (LOX-1), leads to the expression of adhesion molecules such as intracellular adhesion molecule (ICAM), which is responsible for the monocytes adhesion to the endothelium [9,10,12,14-16]. Once the monocytes are in the endothelium, they migrate to the intima (an inner layer of the artery), due to the action of the monocyte chemoattractant protein-1 (MCP-1) [10]. The interaction of ox-LDL and its receptor, leads to an increase of reactive oxygen species (ROS) and an increment on the activity of matrix metalloproteinase (MMP) [4]. Overexpression of MMP is related with some chronic diseases such as inflammation and atherosclerosis [17] and the increase of ROS inactivates the endothelium nitric oxide synthase (eNOS) [5]. All these steps result in the plaque destabilization and rupture, leading to thrombus formation [4].

Amaranth (*Amaranthus hypochondriacus*), a pseudocereal, is an option as a source of proteins that produce bioactive peptides that prevent chronic diseases. The protein content of amaranth is higher (13-19%) than most cereals [18]. It also has bioactive compounds, with health promotion and prevention of CVD and hypercholesterolemia [18,19]. Peptides derived from unprocessed amaranth have antioxidant capacity [20]; antihypertensive, anticarcinogenic and antidiabetic potential [21,22]. Bioactive peptides from whole cereal grains could prevent CVD [23].

Extrusion, a high temperature–short time technology, has been used to obtain pre-cooked flours with high nutritional value [24], antioxidant capacity and anti-inflammatory activity [25,26]. Montoya-Rodríguez et al. [26] reported, in extruded amaranth flour, peptides with active sequences and potential antithrombotic activity. However, no studies have been reported on the effect of extrusion on the anti-atherosclerotic potential of amaranth hydrolysates in human macrophages. Therefore, the aim of this study was to compare the anti-atherosclerotic potential of pepsin-pancreatin hydrolysates from unprocessed (UAH) and extruded amaranth (EAH) in THP-1 lipopolysaccharide (LPS)-induced human macrophages and suggest the mechanism of action.

## Results

### Unprocessed and extruded amaranth hydrolysates showed a reduction in the expression of protein markers including interleukins involved in atherosclerosis

**Figure 1:**
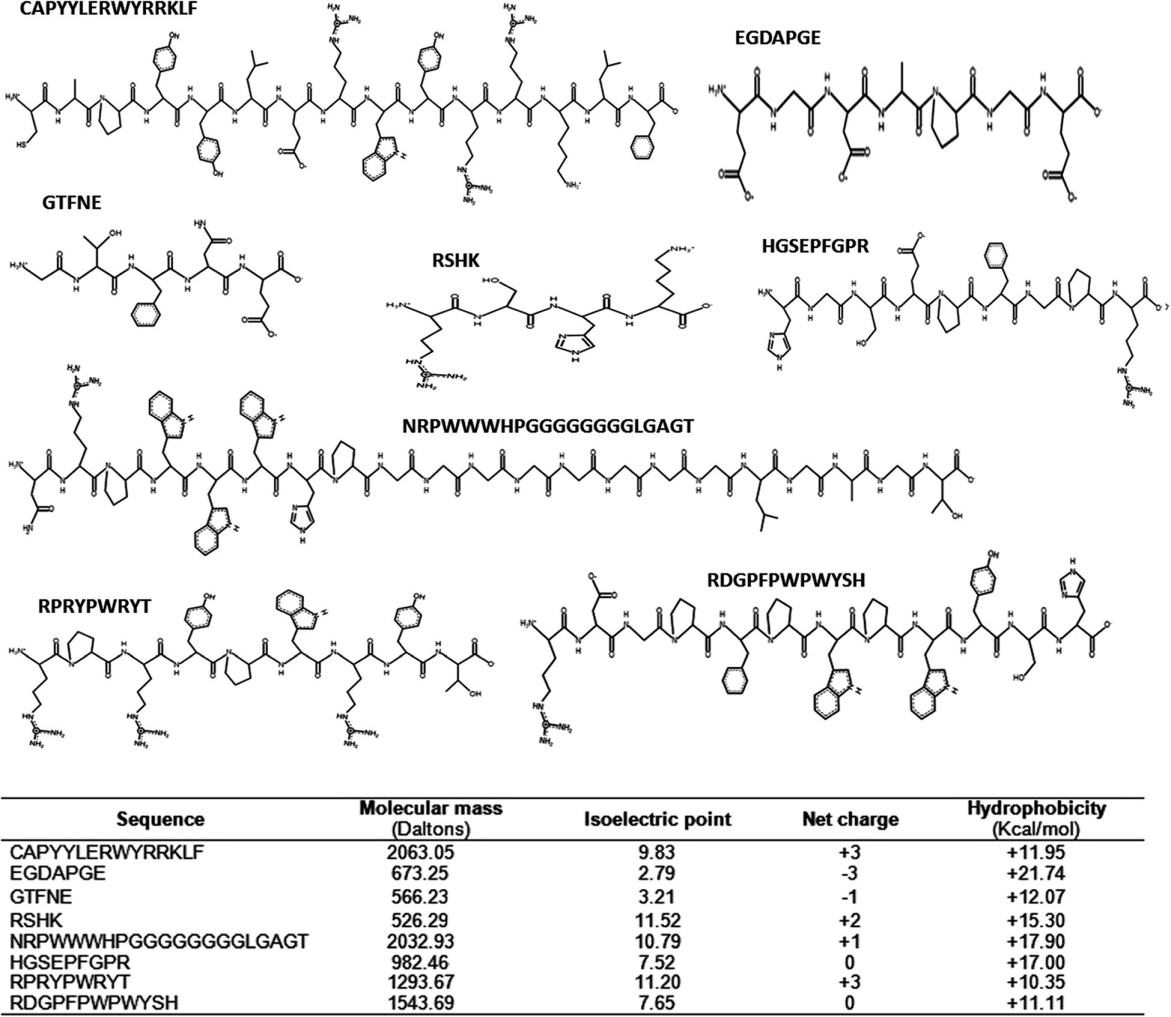
Physicochemical properties of the main peptides found on unprocessed and extruded amaranth hydrolysates using PepDraw tool, including amino acid sequence and structure, molecular mass, net charge, isoelectric point and hydrophobicity.

Figure 1 presents the main peptides found in UAH and EAH including their amino acid sequence and structure, molecular mass, net charge, isoelectric point and hydrophobicity. Table 1 presents some of the proteins involved in the atherosclerosis pathway and the percent reduction found after treatment with UAH and EAH. Table 1 also includes the role of these proteins in the cell. This table highlights the high and significant reduction by both protein hydrolysates on TGF-α which is associated with atherosclerosis; also on IL-4, associated with the secretion of TGF-β; and also on the reduction of IL-32α/β/γ, a cytokine that activates NF-κB.

**Table 1:** **Protein markers involved on inflammatory and atherogenic pathways, their cell action and percent of reduction by UAH and EAH**^**1**^

			**Reduction (%)**	
**Name**	**Full name**	**Cell action**	**UAH**	**EAH**
TGF-α	Transforming growth factor-α	It is associated with vascular pressure and atherosclerosis [27]	100	100
IL-4	Interleukin-4	An increase in repair macrophages (M2) is coupled with secretion of IL-10 and TGF-β [28]	69	100
IL-32α/β/γ	Interleukin-32α/β/γ	Pro-inflammatory cytokine. Responsible of NF-κB activation [29]	100	85
IL-22	Interleukin-22	It has a critical role in modulating local inflammation in certain organs [30]	55	70
IGFBP-3	Insulin-like growth factor-binding protein-3	High levels are related with hypertension and atherosclerosis [31]	71	63
TNF-α	Tumor Necrosis Factor-α	It is expressed by the action of different stimulus, like LPS. It acts as pro-inflammatory stimulus [32]	60	63
GM-CSF	Granulocyte-macrophage colony-stimulating factor	Its function is as a white blood cell growth factor. It is part of the immune/inflammatory cascade [33]	59	63
GRO-α	Chemokine (C-X-C motif) ligand-1	It is a pro-inflammatory chemokine and is secreted by monocytes in response to pro-inflammatory stimuli [34]	62	62
Resistin/ADSF	Adipose tissue-specific secretory factor	It is a pro-inflammatory cytokine [35]	14^*NS*^	62
TfR	Transferrin receptor-1	Involved in the apoptosis of endothelial cells [36]	48	61
GDF-15	Growth differentiation factor 15	It has a role in regulating inflammatory pathway [37]	*NS*	60
IL-11	Interleukin-11	It is rather pro-inflammatory in chronic inflammation [38]	66	57
FGF-19	Fibroblast growth factor-19	Inhibited the hepatic fatty acid synthesis [39]	61	56
MIF	Macrophage migration inhibitory factor	It encodes a lymphokine involved in cell-mediated immunity, immunoregulation and inflammation [40]	46	53
IL-1α	Interleukin-1 α	It can induce the activation of nuclear factor-κB (NF-κB) [41]	41	53
IL-6	Interleukin-6	In chronic inflammation it is rather proinflammatory [42]	64	52
IL-12p70	Interleukin-12p70	It is a key YH-1 cytokine that drives inflammation in numerous models of intestinal inflammation [43]	100	51
PDGF-AA	Platelet-derived growth factor-AA	It has been linked to atherosclerosis [44]	50	49
RANTES	Chemokine(C-C motif) ligand-5	Involved in several clinical inflammatory conditions, such as atherosclerosis [45]	33^*NS*^	47
EMMPRIN	Extracellular-matrix metalloproteinase inducer	It is involved in cytokines activation [46]	*NS*	46
MCP-1	Monocyte-chemo attractant protein-1	It has been linked with chronic inflammatory diseases and atherosclerosis [4,47]	35	42
SDF-1α	Stromal derived cell factor-1α	It can activate the nuclear factor-κB (NF-κB) [48]	19^*NS*^	41
ICAM-1	Intracellular Adhesion Molecule-1	Adhesion of monocytes is mediated by ICAM-1 [4]	*NS*	40
IL-1β	Interleukin-1 β	Low and high levels produced inflammation, resulting in tissue damage and tumor invasiveness [41]	*NS*	40
MMP-9	Matrix Metalloproteinase-9	Over-expression produces some disorders like inflammation and atherosclerosis [17]	*NS*	38
PTX-3	Petraxin-3	Inflammatory marker thought to be more specific to vascular [49]	77	*NS*

^1^Percent of reduction is relative to the positive control. UAH = unprocessed amaranth hydrolysate; EAH = extruded amaranth hydrolysate.

*NS* = not statistically different p <0.05.

**Figure 2:**
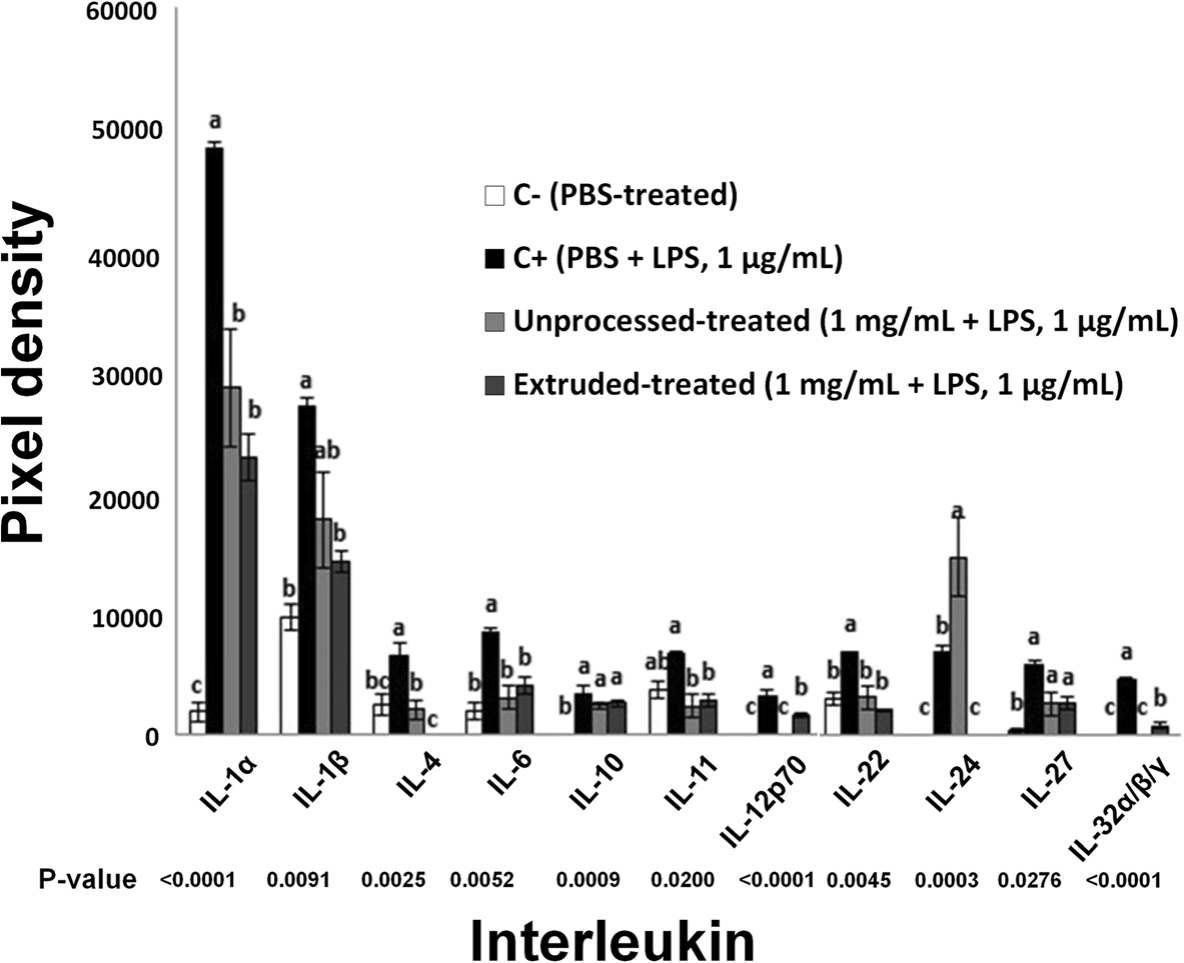
**Effect of unprocessed amaranth hydrolysate and extruded amaranth hydrolysate on human THP-1 macrophages-like cells expression of interleukins related with inflammation and atherosclerosis process.** Bars with different letter means they are statistically different (p < 0.05) relative to the positive control (C+). All treatments contain lipopolysaccharide (LPS, 1 μg/mL) except negative control (C-) treated with PBS-alone.

**Figure 3:**
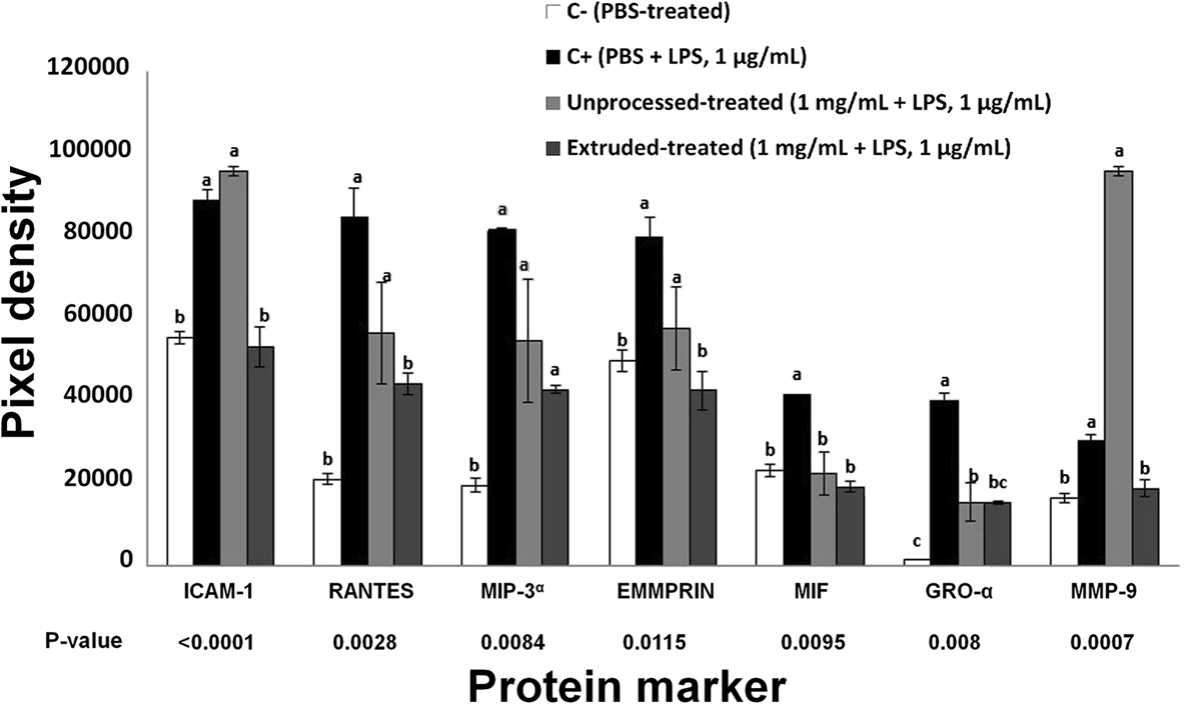
**Effect of unprocessed amaranth hydrolysate and extruded amaranth hydrolysate on human THP-1 macrophages-like cells expression of protein markers related with the process of inflammation and atherosclerosis.** Bars with different letter means they are statistically different (p < 0.05) relative to the positive control (C+). All treatments contain lipopolysaccharide (LPS, 1 μg/mL) except negative control (C-) treated with PBS-alone.

**Figure 4:**
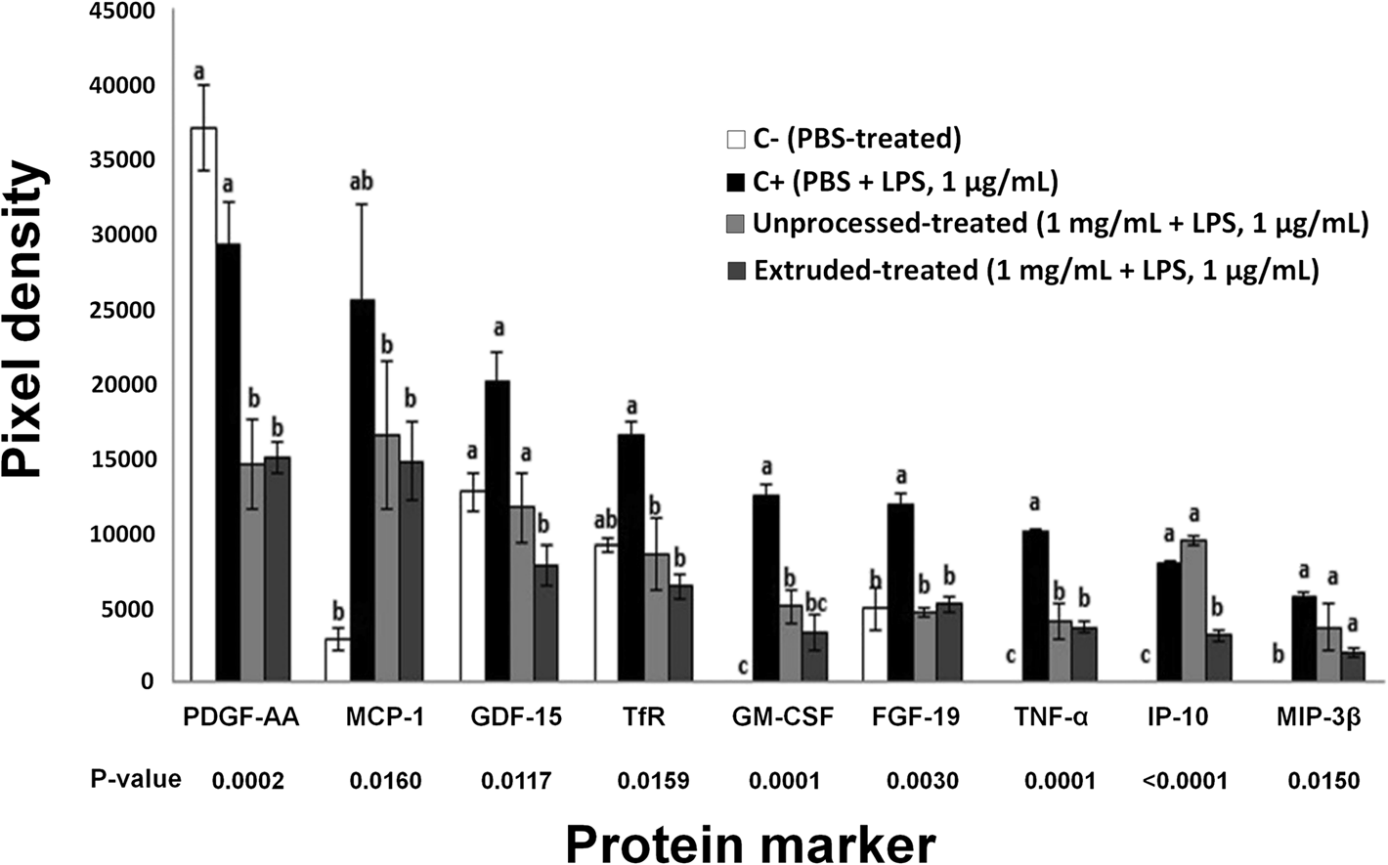
**Effect of unprocessed amaranth hydrolysate and extruded amaranth hydrolysate on human THP-1 macrophages-like cells expression of growth factors related with atherosclerosis.** Bars with different letter means they are statistically different (p < 0.05) relative to the positive control (C+). All treatments contain lipopolysaccharide (LPS, 1 μg/mL) except negative control (C-) treated with PBS-alone.

**Figure 5:**
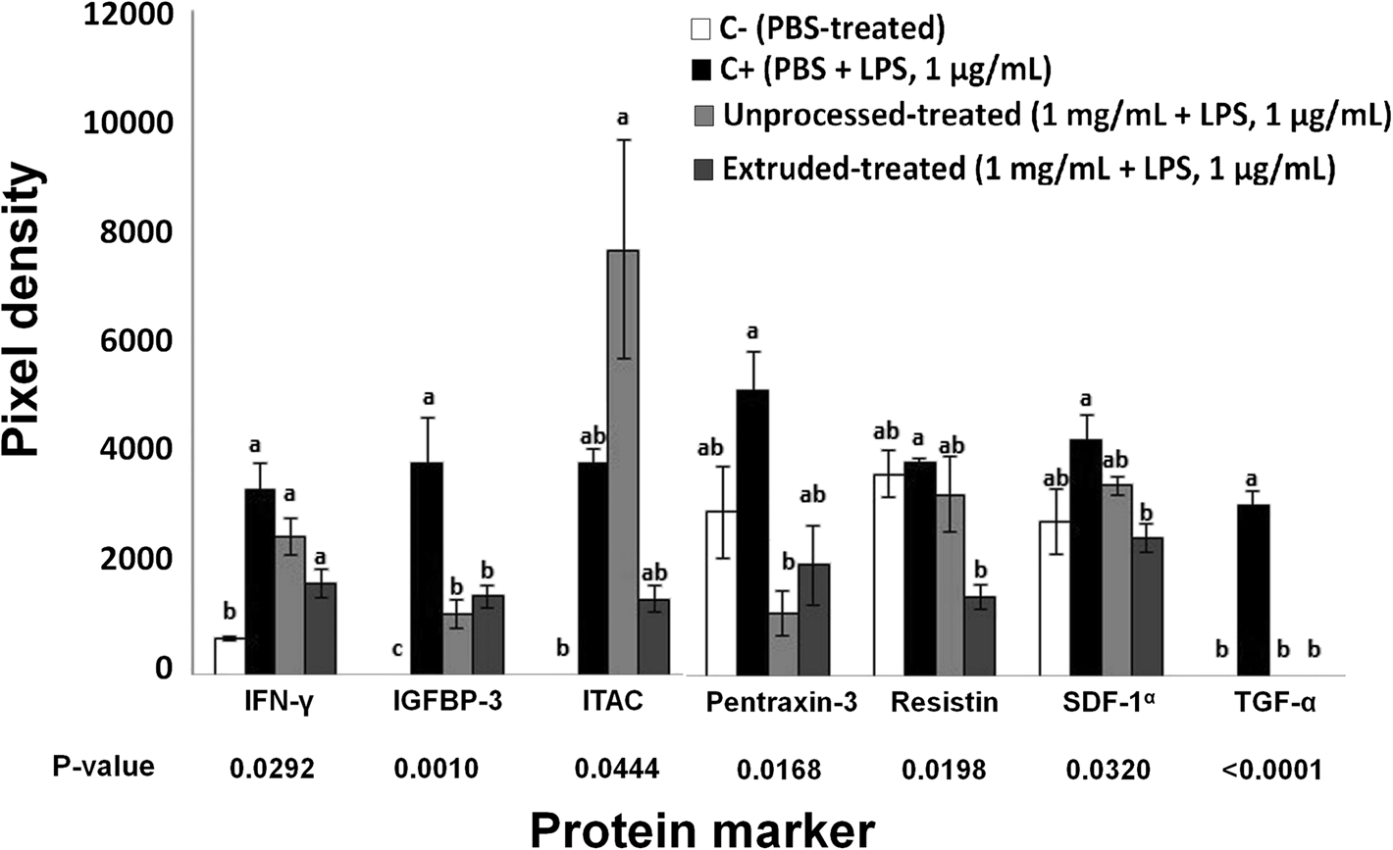
**Effect of unprocessed amaranth hydrolysate and extruded amaranth hydrolysate on human THP-1 macrophages-like cells expression of protein markers related with vascular pressure and atherosclerosis.** Bars with different letter means they are statistically different (p < 0.05) relative to the positive control (C+). All treatments contain lipopolysaccharide (LPS, 1 μg/mL) except negative control (C-) treated with PBS-alone.

Figure 2 shows the comparative effect of UAH and EAH on the expression of interleukins, such as IL-4, IL-6, IL-22 and IL-12p70 related with inflammation and atherosclerosis. UAH and EAH showed a significant (p < 0.05) reduction of 69% and 100% for IL-4; 64% and 52% for IL-6; 55% and 70% for IL-22; 100% and 51% for IL-12p70, respectively. Figure 3 shows the effect of UAH and EAH on the expression of GRO-α, RANTES, ICAM-1 and MMP-9, among other proteins. EAH presented a significant (p < 0.05) reduction of MMP-9 (38%), ICAM-1 (40%) and RANTES (47%); UAH did not show a reduction (p > 0.05) in the expression of these proteins. GRO-α was affected by both UAH and EAH, with a reduction (p < 0.05) of 62% for both. Figure 4 indicates the effect of UAH and EAH on the expression of MCP-1, TfR, GM-CSF, FGF-19, and TNF-α, among other growth factors related with atherosclerosis. UAH and EAH showed a reduction (p < 0.05) of 35% and 42% for MCP-1; 48% and 61% for TfR; 59% and 63% for GM-CSF; 61% and 56% for FGF-19; 60% and 63% for TNF-α, respectively. Figure 5 presents the effect of UAH and EAH on the expression of TGF-α, resistin, and SDF-1α, among other protein markers related with vascular pressure and atherosclerosis. Both, UAH and EAH showed a reduction (p < 0.05) of 100% on the expression of TGF-α. Resistin and SDF-1α were only affected by the action of EAH with a reduction of 62% and 41% (p < 0.05), respectively. Other important molecules which were involved in the atherosclerotic pathway, IL-11 and IL-1α, showed a significant reduction (p < 0.05) in their expression after 24 h of cell treatment with UAH and EAH; EMMPRIN, IL-1β and PTX-3 only showed a significant reduction (p < 0.05) with EAH.

### Extruded amaranth protein hydrolysate reduced the expression of LOX-1, ICAM-1 and MMP-9 in LPS-induced THP-1 macrophage-like human cells

**Figure 6:**
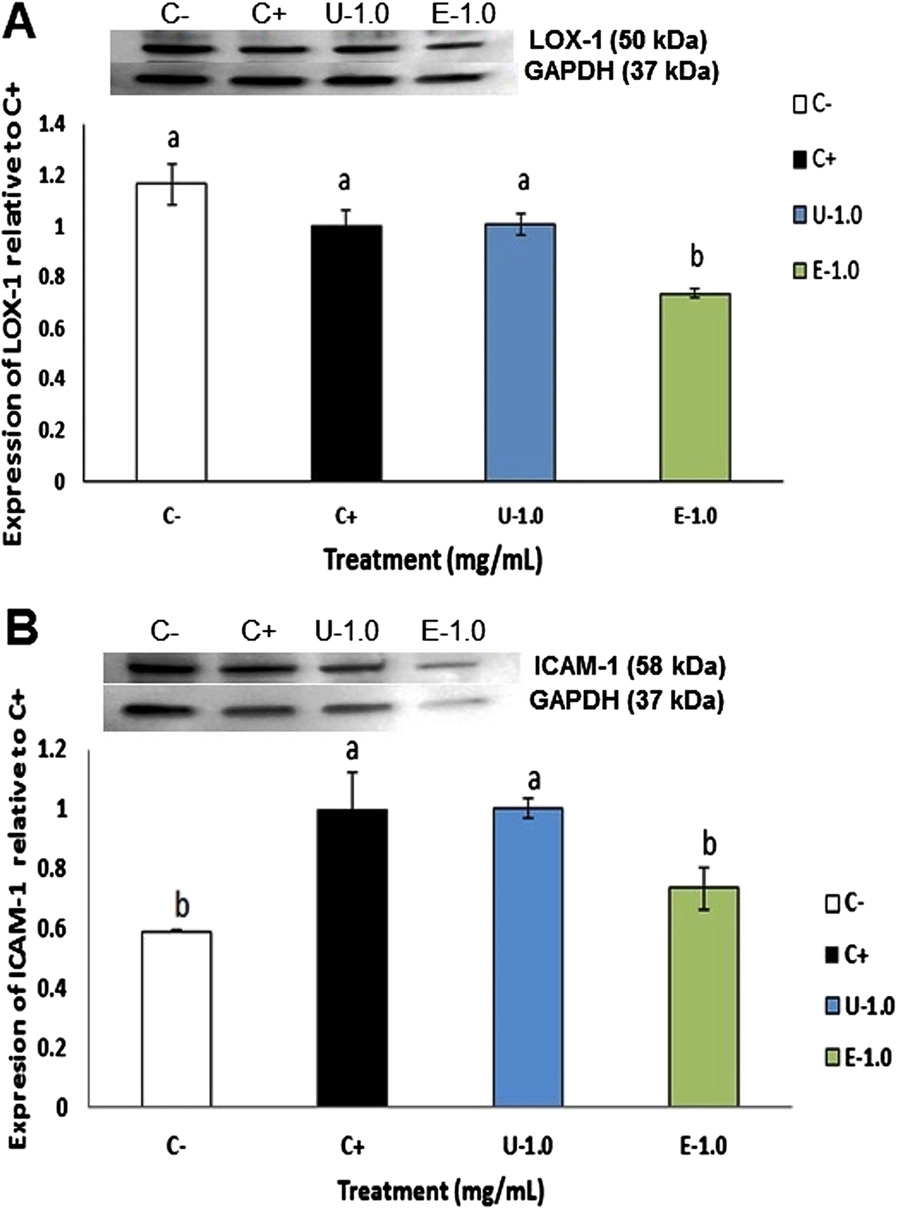
**Effect of extruded amaranth hydrolysates on human THP-1 macrophages-like cells expression of lectin-like oxidized low-density lipoprotein receptor-1 (LOX-1) (A) and on the expression of intracellular adhesion molecule-1 (ICAM-1) (B).** All experiments were performed in at least two independent replicates. Different letter per column means statistically different (p < 0.05) relative to the positive control (C+). All treatments contain lipopolysaccharide (LPS, 1 μg/mL) except negative control (C-) treated with PBS-alone.

**Figure 7:**
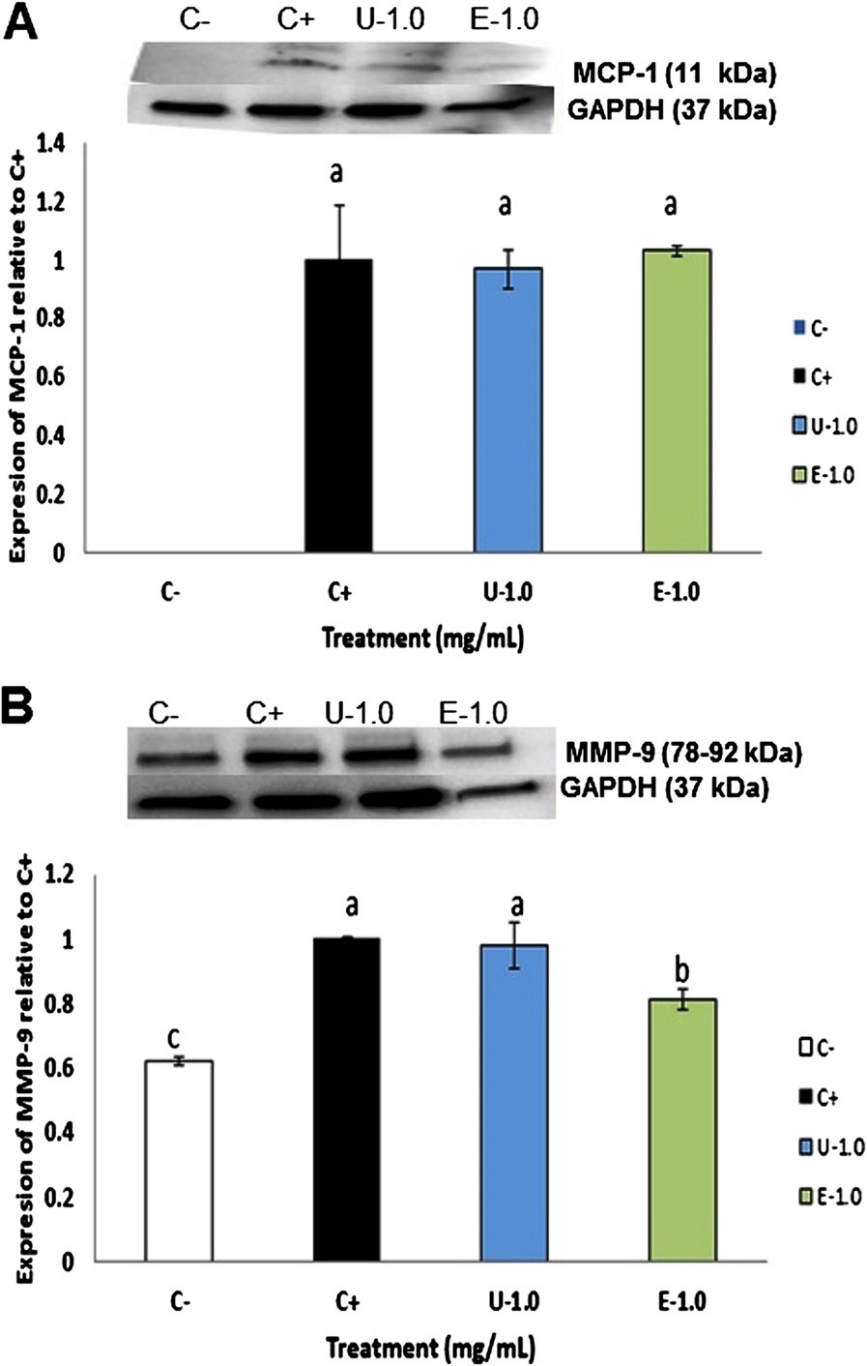
**Effect of extruded amaranth hydrolysates on human THP-1 macrophages-like cells expression of monocyte-chemo attractant protein-1 (MCP-1) (A) and on the expression of matrix metalloproteinase-9 (MMP-9) (B).** All experiments were performed in at least two independent replicates. Different letter per column means statistically different (p < 0.05) relative to the positive control (C+). All treatments contain lipopolysaccharide (LPS, 1 μg/mL) except negative control (C-) treated with PBS-alone.

Figure 6 shows the effect on THP-1 macrophage-like cells from UAH and EAH on the expression of LOX-1 and ICAM-1, molecules involved in the atherosclerotic pathway. LOX-1 expression was significantly reduced (p < 0.05) (27%) at 1 mg/mL treatment with EAH, while no reduction was observed at 1 mg/mL with UAH (Figure 6A). The expression of ICAM-1 was significantly reduced (p < 0.05) (28%) at 1 mg/mL treatment with EAH; UAH did not affect its expression (Figure 6B). MCP-1 did not show a significant difference with the control by western blot (Figure 7A). Figure 7B shows the effect of UAH and EAH in the expression of MMP-9 which was significantly (p < 0.05) reduced by 19% by EAH at 1 mg/mL, while UAH had no effect.

### Extruded amaranth protein hydrolysate reduced the expression of LOX-1, ICAM-1 and MMP-9 molecules in LPS-induced THP-1 macrophage-like human cells by immunocytochemical fluorescence confocal microscopy

**Figure 8:**
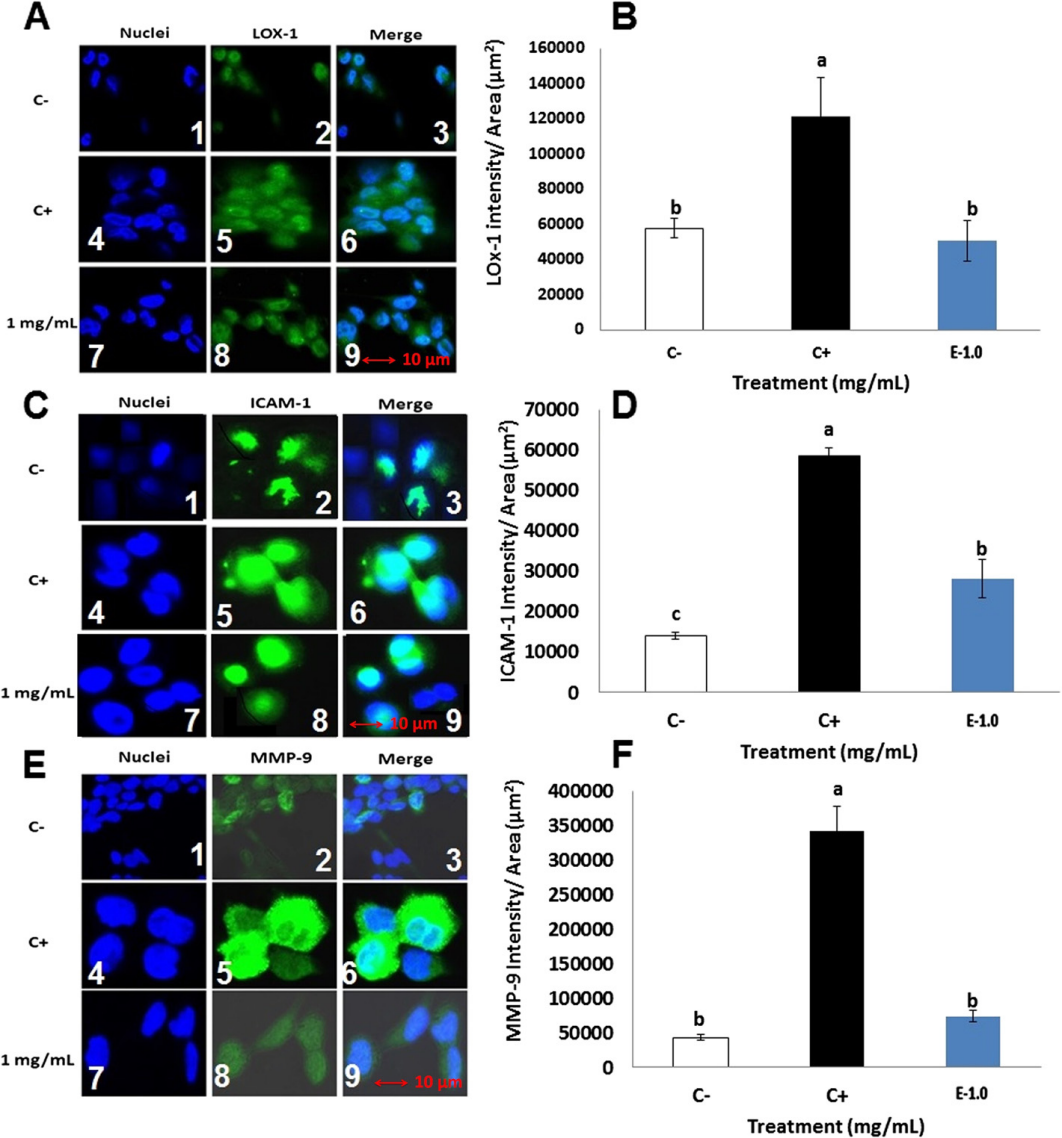
**Confocal laser scanning microscopy depicting two-dimensional immunocytochemical localization of LOX-1 (A), ICAM-1 (C), MMP-9 (E) (green) and nuclei (blue) in human THP-1 macrophages after 24 h of treatment with extruded amaranth hydrolysates.** Quantification of total LOX-1 **(B)**, ICAM-1 **(D)** and MMP-9 **(F)** intensity over their respective area sums over treatment time (μm^2^). (A1) Nuclei C-; (A2) LOX-1 C-; (A3) Merge C-; (A4) Nuclei C+; (A5) LOX-1 C+; (A6) Merge C+; (A7) Nuclei treatment; (A8) LOX-1 treatment; (A9) Merge treatment. (C1) Nuclei C-; (C2) ICAM-1 C-; (C3) Merge C-; (C4) Nuclei C+; (C5) ICAM-1 C+; (C6) Merge C+; (C7) Nuclei treatment; (C8) ICAM-1 treatment; (C9) Merge treatment. (E1) Nuclei C-; (E2) MMP-9 C-; (E3) Merge C-; (E4) Nuclei C+; (E5) MMP-9 C+; (E6) Merge C+; (E7) Nuclei treatment; (E8) MMP-9 treatment; (E9) Merge treatment. Six independent fields of view from two independent cellular replicates were merged together per treatment group. Means with different letters are significantly different from each other (*n* = 2, *p <* 0.05).

Figure 8A and B show the total expression of LOX-1 after treatment of THP-1 cells with EAH. Six independent fields of view from two independent cellular replicates were selected randomly per treatment group. The panel to the left (blue) represents the nuclei, the panel in the middle (green) represents the response to the primary antibody (either LOX-1, ICAM-1 or MMP-9), and the third panel represents the merge of both. Panels 8A1 (nuclei), 8A2 (primary antibody) and 8A3 (merge) show the results after the treatment with PBS alone (C-); 8A4, 8A5 and 8A6 show results after the treatment with PBS plus LPS (C+); while 8A7, 7A8 and 8A9 show the results after the treatment with EAH at 1 mg/mL. Figure 8B summarizes the intensity of LOX-1 after 24 h of treatment with EAH at 1 mg/mL. The intensity of LOX-1 had a significant reduction (p < 0.05) of 58% after 24 h of treatment.

Figures 8C and D show the total expression of ICAM-1. 8C1, 8C2 and 8C3 show the treatment with PBS alone (C-); 8C4, 8C5 and 8C6 show the effect of treatment with PBS plus LPS (C+); while 8C7, 8C8 and 8C9 show only the treatment with EAH at 1 mg/mL. Figure 8D shows the intensity of ICAM-1 after 24 h of treatment with EAH at 1 mg/mL. The intensity of ICAM-1 had a significant reduction (p < 0.05) of 52% after 24 h of treatment. On the other hand Figures 8E and F show the total expression of MMP-9. Panels 8E1, 8E2 and 8E3 show the treatment with PBS alone (C-); 8E4, 8E5 and 8E6 show the treatment with PBS plus LPS (C+); while 8E7, 8E8 and 8E9 show only the treatment with EAH at 1 mg/mL. Figure 8F shows the intensity of MMP-9 after 24 h of treatment with EAH at 1 mg/mL. The intensity of MMP-9 had a significant reduction (p < 0.05) of 79% after 24 h of treatment.

**Figure 9:**
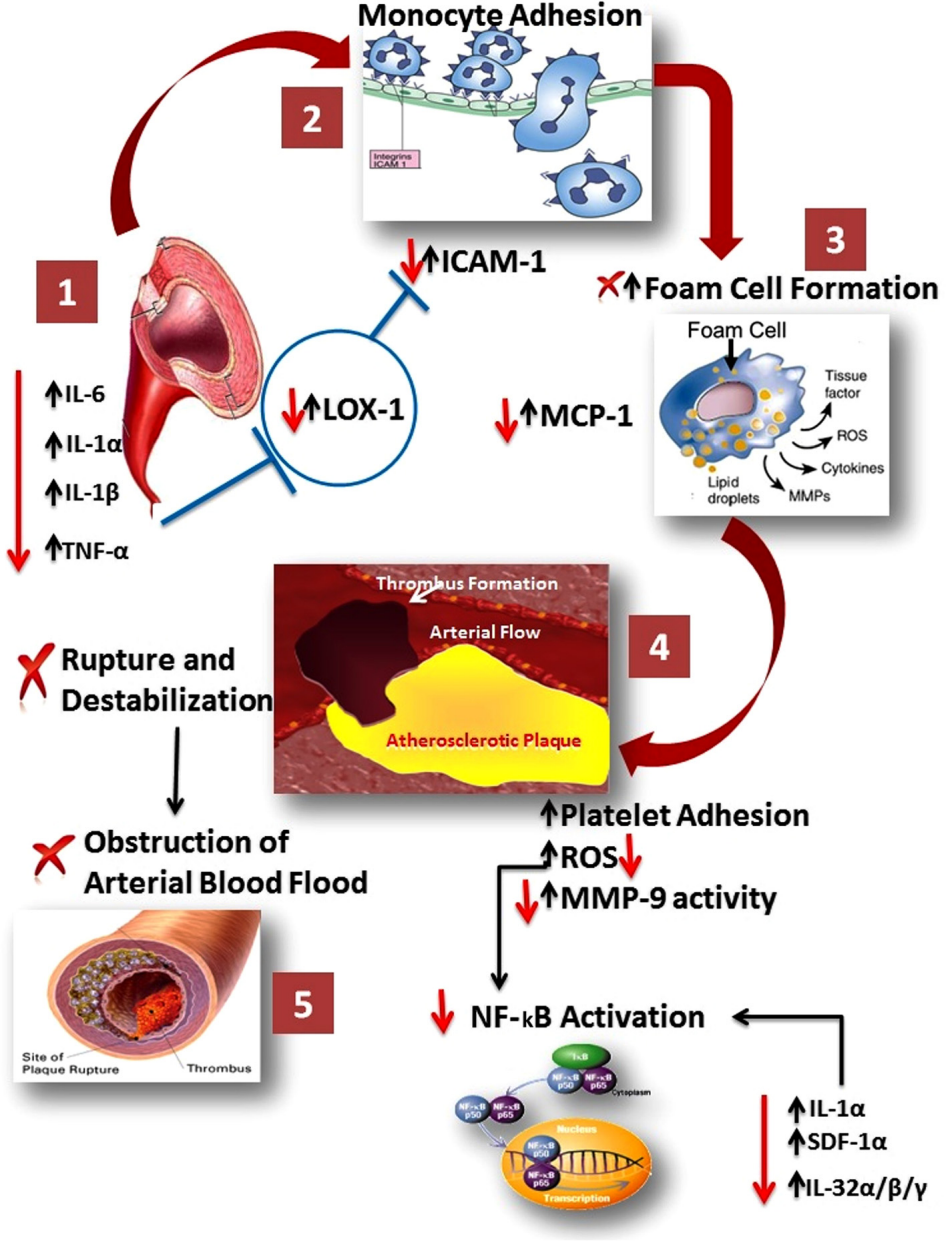
**Potential mechanism of action of the EAH by reducing expression of proteins associated with LOX-1 signaling pathway.** The red arrows indicate the modulation obtained in this study, for each marker, due to EAH treatment. Numbers 1, 2, 3, 4 and 5 indicate the stages of the atherosclerosis process.

Figure 9 indicates with red arrows the reducing expression of proteins by EAH treatment in a suggested potential mechanism of action associated with LOX-1 signaling pathway.

## Discussion

Peptides with an active sequence related to the prevention of atherosclerosis were found in the amaranth protein hydrolysates produced mimicking gastrointestinal digestion after the extrusion of amaranth. In the present study, we observed a reduction in the expression of proteins linked to inflammation and atherosclerosis. IL-6 is a molecule produced at the site of inflammation and when chronic inflammation occurs, it acts as pro-inflammatory marker [42]. Also, when protease-activated receptor (PAR) is activated, it promotes the expression of IL-6, leading to thrombosis [9]. Another molecule that was reduced by the action of the amaranth treatment was chemokine CXC motif ligand-1 (GRO-α), which is a pro-inflammatory chemokine secreted by monocytes in response to pro-inflammatory stimuli like LPS [34]. Other important molecule involved in the activation of NF-κB pathway is IL-1α [41], which was reduced by the action of the treatment with both UAH and EAH. TNF-α plays an important role in the inflammation/atherosclerosis cascade, it acts as pro-inflammatory marker [32]. All of these markers are very important in inflammation and due to the strong relationship between inflammation and atherosclerosis, these markers are also a target for prevention.

The anti-atherosclerotic effect of EAH on LPS-induced human THP-1 macrophages-like could be explained by the capability of the peptides in amaranth protein hydrolysates to inhibit the activation of LOX-1, the principal receptor of ox-LDL in endothelial cells. Its expression is elevated during initial and advanced atherosclerotic lesions as it is capable of binding products induced by inflammation [16,50]. The interaction of ox-LDL with its receptor LOX-1, induced monocytes adhesion to the endothelium via expression of both intracellular adhesion molecule-1 and vascular adhesion molecule-1 (ICAM-1 and VCAM-1) [9]. The activation of adhesion molecules results in the expression of MCP-1, promoting the monocyte migration to the intima [13]. MCP-1 plays an important role in atherogenesis [47]. Using the protein expression array, MCP-1 showed a reduction in its expression in cells treated with both UAH and EAH, while western blot did not detect differences for MCP-1, ELISA method was more sensitive to detect differences in this response.

This molecule-receptor interaction, suggest a transformation of the cells into foam cells [50]. Also, the upregulation of LOX-1 may contribute to the plaque instability. In the present study we observed that at 1 mg/mL, EAH inhibited the activation of LOX-1, ICAM-1 and MMP-9, and a reduction in the expression of MCP-1. The reduction in the expression of these pro-atherosclerotic markers could be explained by the reduction in pro-inflammatory cytokines such as TNFα, IL-6, IL-1α, IL-1β and TGF-α among others, which are stimuli that activated the expression of LOX-1, the principal receptor involved in atherosclerosis pathway [51]. All of these results were confirmed using confocal microscopy, where the effects of the treatment with EAH highly reduced the expression of MMP-9, which is at the end stage of atherosclerosis [4]. The effect observed in the principal markers are in agreement with previous reports, where the positive effect on atherosclerosis prevention has been highlighted if LOX-1 expression is reduced [4,8,21,51]. Previous studies showed that tryptic digest of glutelins from amaranth possessed antihypertensive effect and induced endothelial NO production, resulting in a vasodilatation [21].

Figure 9 shows the suggested mechanism of action associated with LOX-1 signaling pathway. LOX-1 signaling starts (1) by the action of different stimulus such as IL-6, IL1α, TNF-α and LPS [51]. This stimulus produces interaction of the oxidized low density lipoprotein (ox-LDL) with its receptor, LOX-1. This triggers reactions such as the activation of adhesion molecules like ICAM-1, leading into monocyte adhesion (2), resulting in foam cell formation (3), which increases the activity of MCP-1, producing an increase on platelet adhesion (4), reactive oxygen species (ROS) and increase on MMP-9 activity. All these steps produce thrombus formation (5) with a possible rupture and obstruction of the arterial blood flood [4]. Also, the increase in ROS produce the activation of NF-κB, likewise some other markers such as IL-1α, SDF-α and IL-32/α/β/γ also activate NF-κB [27,43]. NF-κB is a family of transcription factors involved in many pathways including inflammation [26]. In our previous work [26] we found peptides from amaranth hydrolysates with potential antioxidant capacity and anti-inflammatory activity. Amaranth hydrolysates inhibited LPS-induced inflammation in human and mouse macrophages by preventing activation of signaling via inhibition of the NF-κB subunits, p-50 and p-65.

## Conclusion

Extruded amaranth protein hydrolysate inhibited LPS-induced markers of atherosclerosis in human THP-1 macrophages-like by preventing the activation of LOX-1 signaling. Furthermore, extrusion improved the anti-atherosclerotic effect of amaranth protein hydrolysates in THP-1 human cells, perhaps attributed to the formation of bioactive peptides during the extrusion process. This is the first study carried out with amaranth protein hydrolysates in search for the potential prevention of cardiovascular disease. Research is underway in our laboratory to test synthesized peptides of interest to evaluate their direct effect, or synergistic effect, of all peptides present in amaranth protein hydrolysates on markers of atherosclerosis.

## Materials and methods

### Material

The amaranth (*Amaranthus hypochondriacus*) grain was grown and harvested during 2011 in Temoac, Morelos, Mexico. Human acute monocytic leukemia cell line (THP-1) and Roswell Park Memorial Institute-1640 media (RPMI-1640, catalogue No. 10–2001), were purchased from American Type Culture Collection (ATCC, Manassas, VA, USA). Fetal bovine serum was purchased from Invitrogen (Grand Island, NY). Streptomycin/penicillin and sodium pyruvate were purchased from Cellgro (Manassas, VA, USA). Lipopolysaccharide from *Escherichia coli* O55:B5 and phorbol 12-myristate 13-acetate (PMA) were purchased from Sigma-Aldrich (St. Louis MO). Lectin-like oxidized low-density lipoprotein receptor-1 (LOX-1) rabbit polyclonal primary antibody (ab-69660) and matrix metalloproteinase-9 (MMP-9) rabbit monoclonal primary antibody (ab-76003) were purchased from Abcam (Cambridge, MA, USA). Monocyte chemoattractant protein-1 (MCP-1) rabbit oligoclonal primary antibody (710002) and intracellular adhesion molecule-1 (ICAM-1) rabbit oligoclonal primary antibody (710278) were purchased from Invitrogen™ (Carlsbad, CA, USA). Glyceraldehyde 3-phosphate dehydrogenase (GAPDH) was purchased from Santa Cruz Biotechnology (Santa Cruz, CA) and anti-mouse and anti-rabbit IgG horseradish peroxidase conjugate secondary antibody were purchased from GE Healthcare. All other chemicals were purchased from Sigma, unless otherwise specified.

### Extrusion process

The extruded amaranth flour was obtained following the procedure reported by Milán-Carrillo *et al.*[25]. The amaranth grains (1 kg lots) were mixed with Ca(OH)_2_ (0.21 g/100 g amaranth) and conditioned with purified water to reach a moisture content of 28%. Each lot was packed in a polyethylene bag and stored at 4°C for 8 h. Before extrusion, the grits were tempered at 25°C for 4 h. A single screw laboratory extruder Model 20 DN (CW Brabender Instruments, Inc., NJ, USA) with a 19 mm screw-diameter; length to diameter 20:1; nominal compression ratio 2:1; and die opening of 3 mm was used. The inner barrel was grooved to ensure zero slip at the wall. The temperature in the barrel was the same for the three zones and the end zone was cooled by air. A third zone, at the die barrel, was not cooled by air. The feed rate was 30 rpm. Extrusion temperature (ET) was defined as temperature at the die end of the barrel. Extrusion operation conditions were: ET, 125°C and screw speed (SS, 130 rpm). The extrudates were cooled, equilibrated at environmental conditions (25°C, RH = 65%), milled (UD Cyclone Sample Mill, UD Corp, Boulder, CO, USA) to pass through an 80-US mesh (0.180 mm) screen, packed in plastic bags, and stored at 4°C until their use.

### Preparation of amaranth protein hydrolysates

The hydrolysis was carried out according to the methodology reported by Megías et al. [52] with some modifications as reported in Montoya-Rodriguez et al. [26]. Briefly, amaranth flour (2.5 g) was suspended in water (1:20 w/v) and a sequential enzyme digestion was carried out with pepsin [EC 3.4.23.1, 662 units/mg; enzyme/substrate, 1:20 (w/w); pH 2.0] and pancreatin [8x USP (a mixture of several digestive enzymes produced by the exocrine cells of the porcine pancreas, EC 232-468-9, Sigma-Aldrich P7545); enzyme/substrate, 1:20 (w/w); pH 7.5] at 37°C for 120 min for each enzyme. The final hydrolysis was stopped by heating at 75°C for 20 min, and the resulting hydrolysate was centrifuged at 20,000 *g* for 15 min at 4°C. The hydrolysates were desalted using 500 Da cellulose acetate membranes (The Nest Group, Inc.), and freeze dried in a Labconco (Kansas, MO) Freeze Dryer 4.5.

### Cell culture and treatments

THP-1 is a human monocytic-derived cell line [53], which matures into human macrophage-like adherent cells following stimulation with phorbol 12-myristate 13-acetate (PMA) [54]. THP-1 was cultured using Roswell Park Memorial Institute-1640 media (RPMI 1640) (ATCC) containing 10% fetal bovine serum (FBS), 1% penicillin-streptomycin, 1% sodium pyruvate and 50 μM β-mercaptoethanol (to prevent crosslinking of Fc receptors on the cell by the antibody in serum and therefore avoiding damaging cell function), and incubated at 37°C in 5% CO_2_/95% air. Phorbol 12-myristate 13-acetate (PMA) was added at a concentration of 162 nM to promote differentiation of THP-1 cells into macrophages as previously described by Furundzija et al. [55], with some modifications. Macrophage differentiation was allowed for 48 h and it was determined by cell morphology and total adhesion to the plate. Human THP-1 macrophages-like were seeded at a density of 1,000,000 cells per 2-mL in a 6-well plate and after complete adhesion, treated with sterile-filtered UAH and EAH at a concentration of 1 mg/mL for 24 h. This concentration was selected based on our preliminary data of the concentration needed to inhibit 50% of the anti-inflammatory pathway (IC_50_) [26]. To stimulate inflammation, cells were treated with 1 μg/mL LPS dissolved in growth medium. Cells treated with PBS and LPS served as positive control while cells treated with PBS-alone served as negative control. After 24 h of treatment, growth medium was aliquot and immediately frozen at -20°C until use.

### Cell viability

Cell viability of THP-1 human macrophages-like was performed following the method reported in our previous research [26]. The viability of the cells was not affected with the highest treatment concentration (1 mg/mL) used in the present study.

### Proteins/cytokines array

The expression profile of atherogenic-related proteins was performed and analyzed using a Human XL Cytokine Array (Ary022, R&D Systems, MN) according to the manufacturer instructions. Briefly, THP-1 cells were seeded at a density of 1 × 10^6^ cells in a 75 cm^2^ canted neck flask, with PMA at 162 nM (to promote differentiation) for 24 h at 37°C in 5% CO_2_/95% air. After 24 h, cells were washed twice with PBS to remove the PMA and treated with PBS, UAH and EAH (1 mg/mL) and LPS for 24 h. Cells treated with PBS alone was used as a negative control. After 24 h of treatment, cells were harvested using lysis buffer reagent and the expression of proteins in the cell lysates was determined following the manufacturer’s protocol.

### Western blot analysis of LOX-1, ICAM-1 and MMP-9

Cell lysates obtained in a similar manner as for the protein array were used for this study. Protein concentration of the whole cell lysates was determined by protein DC assay (Biorad, Hercules, CA) and equal volume of the lysates and Laemmli buffer containing 5% β-mercaptoethanol was boiled for 5 min. Cell lysates were immediately frozen until used for western blotting to measure expression of pro-atherosclerosis markers such as LOX-1, ICAM-1 and MMP-9. Equal amount of protein (25 μg) from whole cell lysates was loaded in 4–20% gradient SDS-polyacrylamide gels (Bio-Rad Laboratories, Inc.). The separated proteins were transferred onto PVDF membranes (GE Healthcare Biosciences) and blocked with 5% nonfat dry milk in 0.1% Tris-buffered saline Tween 20 (TBST) for 1 h at 4°C. The membranes were washed with 0.1% TBST and incubated with either LOX-1 rabbit polyclonal primary antibody, ICAM-1 rabbit polyclonal primary antibody or MMP-9 rabbit monoclonal primary antibody overnight at 4°C. The membranes were washed again with TBST (5x, 5 min each) and incubated with anti-rabbit IgG horseradish peroxidase conjugate secondary antibody (GE Healthcare, Piscataway, NJ) for 1 h at room temperature. After incubation and repeated washing, the membranes were prepared for detection using a 1:1 mixture of chemiluminescent reagents A (luminol solution) and B (peroxide solution) (GE Healthcare, Piscataway, NJ). The membrane pictures were taken on a GelLogic 4000 Pro Imaging System (Carestream Health, Inc., Rochester, NY). The relative amount of each target protein was normalized to GAPDH. All western blot procedures were determined in at least three independent trials. Each treatment was performed in at least a triplicate to confirm reproducibility.

### Immunocytochemical fluorescence confocal microscopy

Briefly, 20, 000 THP-1 cells were seeded in 300 μL of phenol red-free RPMI-1640® medium (Life Technologies) with PMA at 162 nM (to promote differentiation) in ibiTreat microscopy chambers (ibidi) for 48 h at 37°C in 5% CO_2_/95% air. After 48 h of differentiation, cells were washed twice with PBS to remove all the PMA and treated with EAH at 1 mg/mL for 24 h. Cells were washed three times with PBS and fixed with 4% paraformaldehyde aqueous solution (Electron Microscopy Sciences) for 30 min at room temperature, washed three times 5 min each with PBS, and permeabilized with 0.1% Triton X-100 in PBS for 15 min at room temperature. Cells were washed once with PBS and incubated with ultra-cold HPLC-grade methanol for 15 min at -20°C. Methanol was removed and replaced with PBS and incubated for 30 min at room temperature. Cells were blocked with Image-iT FX Signal Enhancer (Life Technologies) for 30 min at room temperature, washed once with PBS and incubated with either LOX-1 (5 μg/mL), ICAM-1 (1:250) or MMP-9 (1:250) monoclonal primary antibody for 5 h at 37°C. After incubation, cells were washed three times 5 min each with PBS and incubated with Alexa Fluor 488 Goat Anti-rabbit (Life Technologies) secondary antibody (1:200) for 3 h at 37°C. Cells were washed three times with PBS and cured with ProLong gold antifade reagent with DAPI (Life Technologies) for 24 h at 25°C in the dark. The chamber was stored at 4°C until further use. The cells were visualized using a Carl Zeiss LSM 700 Laser Scanning Microscope (Carl Zeiss AG, Germany) with 63x oil immersion objective. Total intensities and area sums were quantified with AxioVision Rel 4.8 (Carl Zeiss).

### Statistical analysis

Statistical analyses were conducted using the proc GLM procedures of SAS version 9.3 (SAS Inst. Inc., Cary, NC). Group mean comparisons were conducted using LSD means and were considered to be significant at p < 0.05 based on minimum significant differences from one-way analysis of variance (ANOVA) with alpha ≤ 0.05. All analyses were performed in at least three independent replicates.

## Abbreviations

ATCC: American type culture collection; CVD: Cardiovascular disease; Da: Daltons; DAPI: 4′,6-diamidino-2-phenylindole (fluorescent stain); EAH: Extruded amaranth hydrolysates; ELISA: Enzyme-linked immunosorbent assay; EMMPRIN: Extracellular-matrix metalloproteinase inducer; eNOS: Endothelium nitric oxide synthase; ET: Extrusion temperature; FBS: Fetal bovine serum; FGF-19: Fibroblast growth factor-19; GAPDH: Glyceraldehyde 3-phosphate dehydrogenase; GM-CSF: Granulocyte-macrophage colony-stimulating factor; GRO-α: Chemokine (C-X-C motif) ligand-1; HPLC: High-performance liquid chromatography; ICAM-1: Intracellular Adhesion Molecule-1; IL-1α: Interleukin-1α; IL-1β: Interleukin-1β; IL-4: Interleukin-4; IL-6: Interleukin-6; IL-11: Interleukin-11; IL-12p70: Interleukin-12p70; IL-22: Interleukin-22; IL-32α/β/γ: Interleukin-32α/β/γ; LDLs: Low density lipoproteins; LOX-1: lectin-like oxidized low-density lipoprotein receptor-1; LPS: Lipopolysaccharide; MCP-1: monocyte chemoattractant protein-1; MMP: Matrix metalloproteinase; MMP-9: Matrix Metalloproteinase-9; NF-κB: nuclear factor kappa-light-chain-enhancer of activated B cells; NO: Nitric oxide; ox-LDL: Oxidized low density lipoprotein; PAR: protease-activated receptor; PBS: Phosphate buffer saline; PMA: phorbol 12-myristate 13-acetate; PTX-3: Petraxin-3; PVDF: Polyvinylidene fluoride; RANTES: Chemokine(C-C motif) ligand-5; RH: Relative humidity; ROS: Reactive oxygen species; RPMI-1640: Roswell park memorial Institute-1640 media; SDF-1α: Stromal derived cell factor-1α; SDS: Sodium dodecyl sulfate; SS: Screw speed; TBST: Tris buffer saline tween 20; TfR-1: Transferrin receptor-1; TGF-α: Transforming growth factor-α; TGF-β: Transforming growth factor-β; THP-1: human monocytic-derived cell line; TNF-α: Tumor Necrosis Factor-α; UAH: Unprocessed amaranth hydrolysates; VCAM-1: Vascular adhesion molecule-1.

## Competing interests

The authors declared that they do not have competing interests.

## Authors’ contributions

J M-C and C R-M proposed the project. A M-R designed and performed the experiments and wrote the manuscript. V P.D and A M-R performed the data analysis. E G de M provided guidance throughout the research and revised the manuscript. All authors read and approved the manuscript.
